# The Refinement of Genetic Predictors of Multiple Sclerosis

**DOI:** 10.1371/journal.pone.0096578

**Published:** 2014-05-02

**Authors:** Giulio Disanto, Ruth Dobson, Julia Pakpoor, Ramyiadarsini I. Elangovan, Rocco Adiutori, Jens Kuhle, Gavin Giovannoni

**Affiliations:** 1 Queen Mary University of London, Blizard Institute, Barts and The London School of Medicine and Dentistry, London, United Kingdom; 2 Oxford medical school, University of Oxford, John Radcliffe Hospital, Oxford, United Kingdom; Institute Biomedical Research August Pi Sunyer (IDIBAPS) - Hospital Clinic of Barcelona, Spain

## Abstract

A recent genome wide association study (GWAS) demonstrated that more than 100 genetic variants influence the risk of multiple sclerosis (MS). We investigated what proportion of the general population can be considered at high genetic risk of MS, whether genetic information can be used to predict disease development and how the recently found genetic associations have influenced these estimates. We used summary statistics from GWAS in MS to estimate the distribution of risk within a large simulated general population. We profiled MS associated loci in 70 MS patients and 79 healthy controls (HC) and assessed their position within the distribution of risk in the simulated population. The predictive performance of a weighted genetic risk score (wGRS) on disease status was investigated using receiver operating characteristic statistics. When all known variants were considered, 40.8% of the general population was predicted to be at reduced risk, 49% at average, 6.9% at elevated and 3.3% at high risk of MS. Fifty percent of MS patients were at either reduced or average risk of disease. However, they showed a significantly higher wGRS than HC (median 13.47 vs 12.46, *p* = 4.08×10^−10^). The predictive performance of the model including all currently known MS associations (area under the curve = 79.7%, 95%CI = 72.4%–87.0%) was higher than that of models considering previously known associations. Despite this, considering the relatively low prevalence of MS, the positive predictive value was below 1%. The increasing number of known associated genetic variants is improving our ability to predict the development of MS. This is still unlikely to be clinically useful but a more complete understanding of the complexity underlying MS aetiology and the inclusion of environmental risk factors will aid future attempts of disease prediction.

## Introduction

Multiple sclerosis (MS) is a complex disorder of the central nervous system with a strong genetic component [1]. Indeed, the risk of developing this condition in biological relatives of MS patients increases with increasing degree of kinship; this observation has provided the rationale for genetic studies in MS [2].

The main genetic locus for MS (the HLA-DRB1*1501 allele) was discovered in the 1970s within the major histocompatibility complex (MHC) region, long before the era of genome wide association studies (GWAS) [3]. However, the genetic role in MS susceptibility is not limited to the MHC, and the development of GWAS has provided further insights into MS genetics. Two studies published in 2011 by the International Multiple Sclerosis Genetics Consortium (IMSGC) and the Wellcome Trust Case Control Consortium 2 provided evidence for approximately 60 single nucleotide polymorphisms (SNPs) located outside the MHC influencing MS risk [4], [5]. More recently, the IMSGC performed an updated GWAS that included almost 30,000 MS patients and used a novel SNP array (the ImmunoChip, (Illumina INC, USA)) specifically designed for immune mediated diseases such as MS. This study was able to further increase the number of known MS associated variants to 110 [6].

The identification of individuals carrying a considerably high genetic risk of MS in the general population could be relevant for the development of disease prevention strategies and holds major public health implications. However, it remains unclear what proportion of the general population can be considered at substantially increased risk of disease and whether we will ever be able to predict MS development based on genetic information alone. Previous attempts to do so using GWAS data have shown that genetics is only moderately able to discriminate between MS patients and healthy individuals and that this is still far from being useful clinically [7]–[9]. We aimed to investigate whether the predictive performance of MS genetic associations has changed given that their number has now exceeded the remarkable threshold of 100.

## Methods

We extracted summary statistics (odds ratios (OR) and risk allele frequencies) for all currently known MS associations located outside the MHC from the recent ImmunoChip based GWAS performed by the IMSGC [6]. This article did not report association results within the MHC and therefore summary statistics regarding the *HLA-DRB1* association were extracted from a previous meta-analysis also published by the IMSGC in 2011 [5].

The R package “REGENT” (Risk Estimation for Genetic and Environmental Traits) was used to estimate the proportion of population predicted to be at reduced, average, elevated and high risk of MS under three separate models considering: 1) only the *HLA-DRB1* association; 2) *HLA-DRB1* + associations known in 2011 (Old SNPs) (n = 62) [4], [5]; 3) *HLA-DRB1* + all currently known associations (full model, n = 110) (Table S1) [6]. REGENT applies summary GWAS statistics to a large simulated population. In particular, the distribution of genotypes across 100,000 hypothetical individuals is simulated based on the allelic frequencies reported in published GWAS assuming the presence of Hardy-Weinberg Equilibrium. Secondly, the odds ratios reported for each SNP and the sample size used in the original association study are used to calculate the overall risk of each individual profile with 95% confidence intervals (CI) assuming a multiplicative model between alleles. These estimates of risk are scaled by the risk profile which is closest to the mean risk of the simulated population (baseline risk) to calculate an individual relative risk (RR) [10], [11]. All individuals with 95%CI overlapping with the 95%CI of the baseline risk profile are defined at average risk (i.e. their risk is not significantly different from the baseline risk). The remaining individuals are also grouped into additional risk categories (reduced, elevated and high) based on their 95%CI (i.e. individuals at reduced and elevated risk of disease are those whose 95%CI lie below and above the baseline profile 95%CI respectively; individuals at high risk are those whose 95%CI lie above the 95%CI of the first elevated risk profile) [10], [11].

Blood was drawn, DNA extracted and MS associated variants profiled using the ImmunoChip (Illumina INC, USA) in a total of 73 MS patients and 99 ethnically matched healthy controls (HC) recruited at the Blizard Institute, Queen Mary University (Whitechapel, London, United Kingdom). Allele frequencies, missing genotypes and Hardy-Weinberg equilibrium tests for each investigated SNP are reported in Table S2. After excluding those samples with more than 10% missing genotypes, 70 MS and 79 HC were used for analysis. We used REGENT to estimate how many MS and HC were included in each category of risk under each model [10]. Furthermore, a weighted genetic risk score (wGRS) was calculated for each individual as described in De Jager et al (i.e. by multiplying the number of risk alleles by the weight of each SNP and then taking the sum across all associations considered) [7]. The ability of this estimate of risk to predict MS status was assessed using receiver operating characteristic (ROC) curve. Differences in the predictive performance of different genetic models were tested using the DeLong’s test for correlated ROC curves in the R package “pROC”. This study was conducted according to the principles expressed in the Declaration of Helsinki and obtained ethical permission (East London REC 1 (ref. 10/H0704/62)). All participants provided their written consent to participate in this study using a standardised consent form approved by the ethics committee. Individual genotypes of MS patients and HC are available on request.

## Results

**Figure 1:**
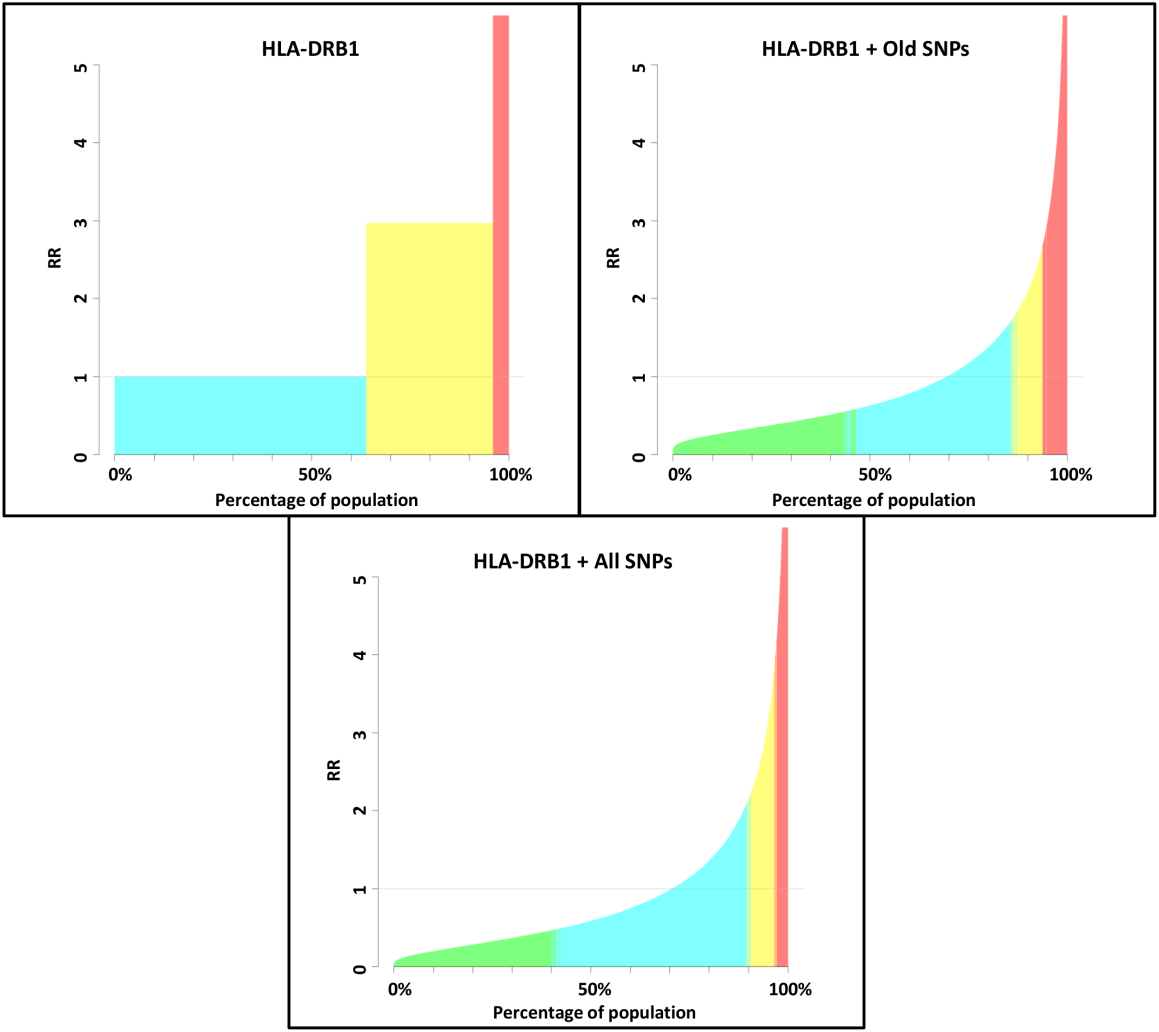
GWAS statistics (OR and risk allele frequencies) were used to simulate a population of 100,000 individuals under different models considering: only HLA-DRB1, HLA-DRB1 + MS associations known in 2011 and HLA-DRB1 + all currently known MS associations. *Categories of risk were defined based on the 95%CI of risk of each individual (see methods). Green = reduced risk, blue = average risk, yellow = elevated risk, red = high risk.*

The distribution of risk across the simulated general population considerably varied based on the genetic variants that were considered in the analysis. For example, when only the *HLA-DRB1* association was considered, all individuals were grouped in three risk categories (average, elevated and high) corresponding to their *HLA-DRB1* status (homozygous DRB1*15 negative, DRB1*15 heterozygous and homozygous DRB1*15 positive) (figure 1, table 1). This is because only these three genotypes are possible at this locus and based on the allelic frequency of the MS associated *HLA-DRB1* allele, the baseline risk used as reference to calculate risk categories is the one of homozygous negative individuals.

**Table 1:** Proportion of population and 95%CIs for each category of risk considering only HLA-DRB1, HLA-DRB1 + MS associations known in 2011 and HLA-DRB1 + all currently known MS associations.

Model considered		Risk Category			
		Reduced	Average	Elevated	High
*HLA-DRB1*	95% CI	0–0.89	0.89–1.12	1.12–3.27	3.27–Inf
	Proportion of population	0.0%	63.8%	32.1%	4.1%
*HLA-DRB1* + Old associations	95% CI	0–0.76	0.76–1.34	1.34–2.06	2.06–Inf
	Proportion of population	44.8%	41.6%	7.5%	6.1%
*HLA-DRB1* + All associations	95% CI	0–0.70	0.70–1.47	1.47–2.71	2.71–Inf
	Proportion of population	40.8%	49.0%	6.9%	3.3%

In brief, GWAS statistics (OR and risk allele frequencies) are used to simulate a population of 100,000 individuals. An overall genetic risk of MS is calculated for each individual and scaled by the mean risk profile. Categories of risk are defined based on the 95%CI of risk of each individual (see methods).

When old associated SNPs were included in the model, the RR of MS appeared more continuously distributed across the population and the proportion of individuals at reduced, average, elevated and high risk was 44.8%, 41.6%, 7.5% and 6.1% respectively (figure 1, table 1). Including the more recent associations did not substantially change these estimates and a substantial proportion of population (40.8%) was still predicted to be at reduced (RR 95%CIs = 0.00–0.70), 49% at average (RR 95%CIs = 0.70–1.47), 6.9% at elevated (RR 95%CIs = 1.47–2.71) and 3.3% at high risk of MS (RR 95%CIs = 2.71–Inf) (figure 1 and table 1).

We then estimated the proportion of our genotyped MS and HC individuals within the risk categories defined by REGENT under each genetic model. We found that the proportion of MS patients identified at elevated and high risk of disease was consistently higher than that of HC. When the full model was considered, 20% and 30% of MS patients vs 15.2% and 8.9% of HC were at elevated and high risk of MS respectively (table 2). However, even when all known variants were included in the model, 50% of MS patients were at either average or reduced risk of disease.

**Table 2:** Proportion of MS patients and HC in different risk categories and median wGRS with interquartile range (IQR) of MS and HC in each model (only HLA-DRB1, HLA-DRB1 + MS associations known in 2011 and HLA-DRB1 + all currently known MS associations).

Model considered	Group	Risk category				Median wGRS (IQR)	Mann-Whitney *p*
		Reduced	Average	Elevated	High		
*HLA-DRB1*	MS	0 (0.0%)	26 (37.1%)	38 (54.3%)	6 (8.6%)	1.09 (0.00–1.09)	4.36×10^−7^
	HC	0 (0.0%)	62 (78.5%)	15 (19.0%)	2 (2.5%)	0.00 (0.00–0.00)	
*HLA-DRB1 + Old associations*	MS	14 (20%)	46 (65.7%)	6 (8.6%)	4 (5.7%)	9.14 (8.39–9.61)	2.14×10^−8^
	HC	46 (58.2%)	31 (39.2%)	2 (2.5%)	0 (0%)	8.15 (7.74–8.68)	
*HLA-DRB1 + All associations*	MS	6 (8.6%)	29 (41.4%)	14 (20.0%)	21 (30.0%)	13.47 (12.88–14.06)	4.08×10^−10^
	HC	13 (16.5%)	47 (59.5%)	12 (15.2%)	7 (8.9%)	12.46 (11.88–12.91)	

In brief, the genotype at MS associated loci was used to assign each individual to the categories of risk identified using the simulated population from table 1. Furthermore, a weighted genetic risk (wGRS) was calculated by multiplying the number of risk alleles by the weight of each SNP and then taking the sum across all associations (see methods).

**Figure 2:**
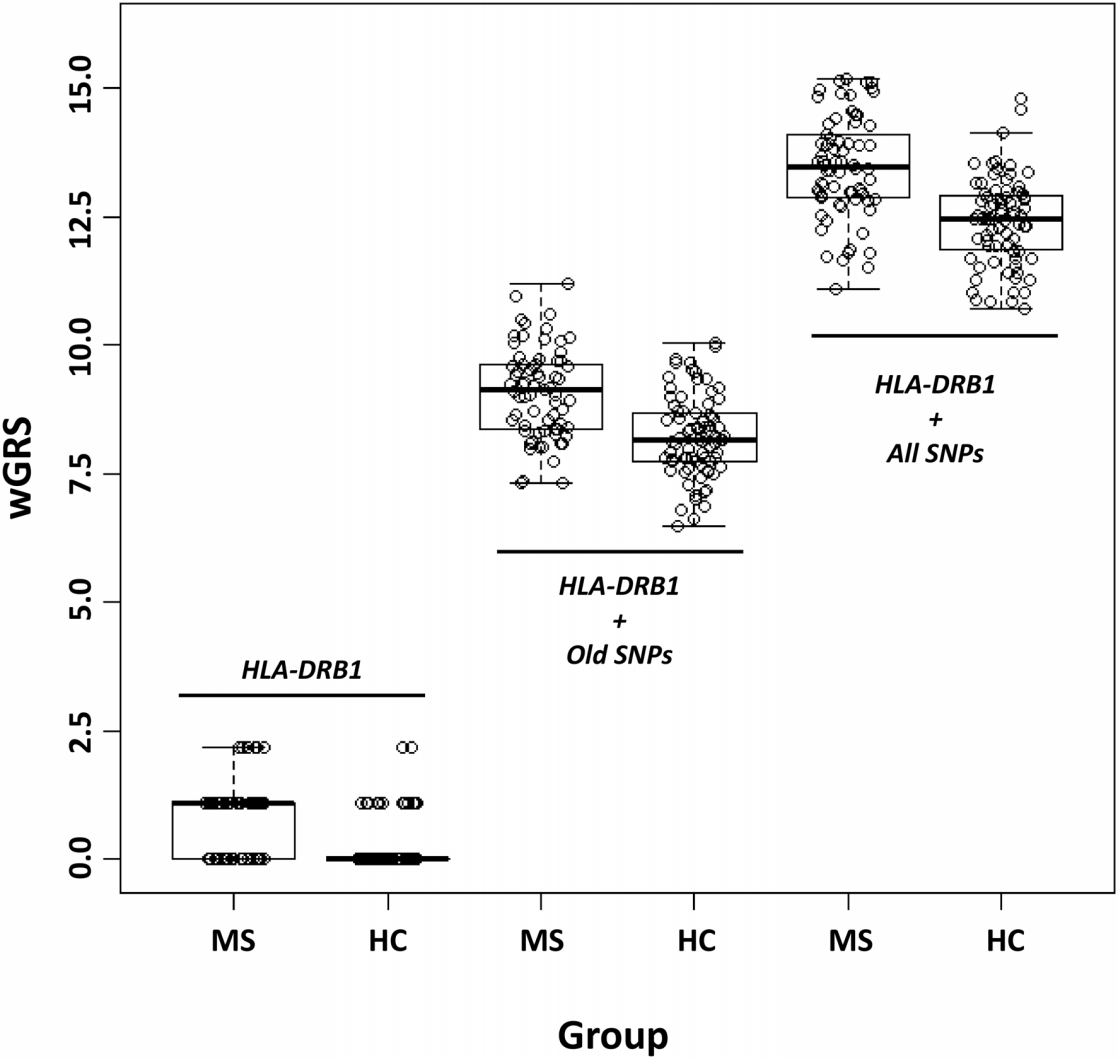
Boxplots of weighted genetic risk score (wGRS) in MS patients and HC considering only HLA-DRB1, HLA-DRB1 + MS associations known in 2011 and HLA-DRB1 + all currently known MS associations. *The wGRS was calculated by multiplying the number of risk alleles by the weight of each SNP and then taking the sum across all associations (see methods). The whiskers extend to the most extreme data point which is no more than 1.5 times the IQR from the box.*

**Figure 3:**
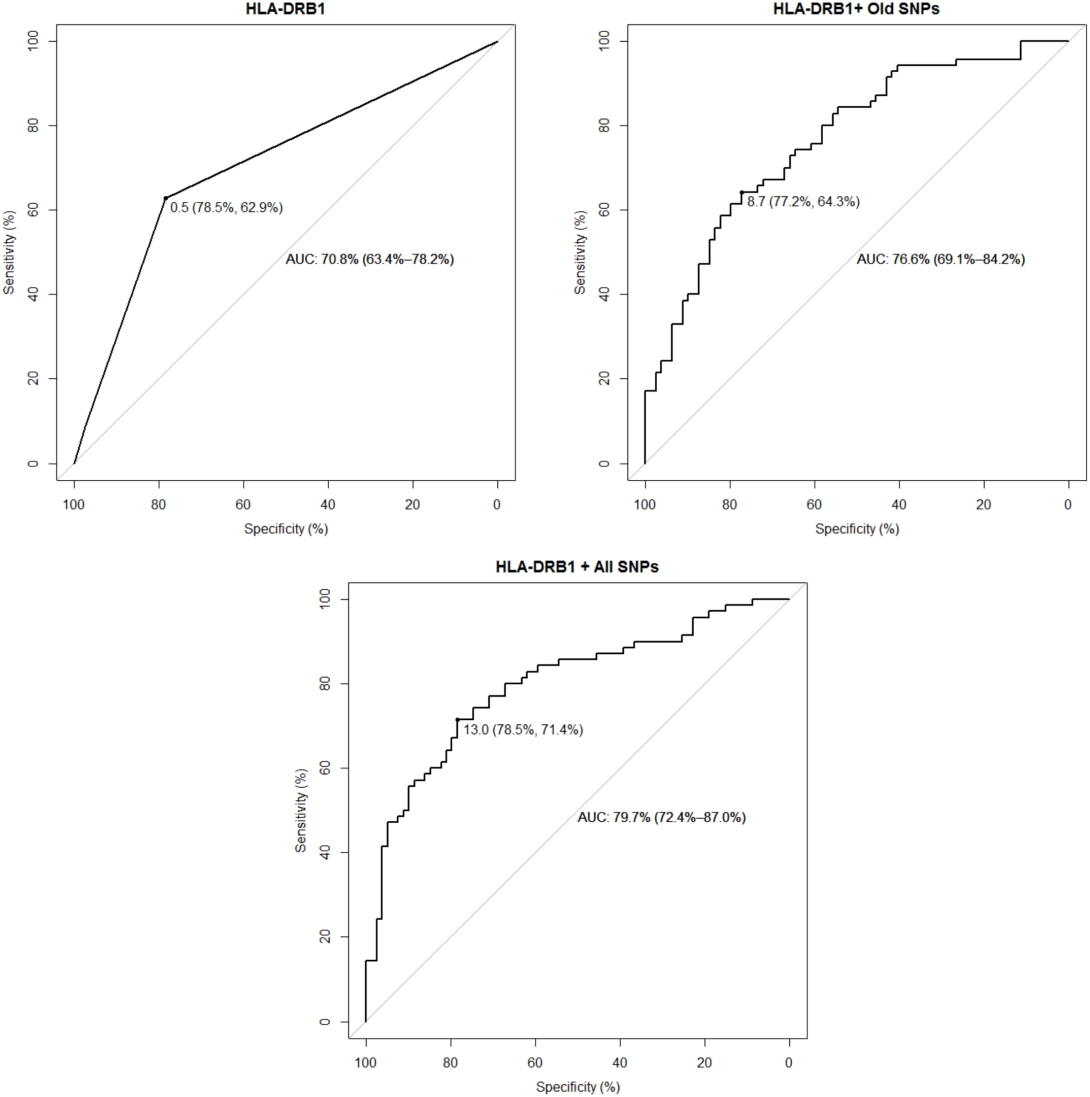
Predictive performance of the wGRS assessed using receiver operating characteristic (ROC) curves when considering only HLA-DRB1, HLA-DRB1 + MS associations known in 2011 and HLA-DRB1 + all currently known MS associations. *The wGRS threshold providing the best predictive performance is also shown (specificity and sensitivity within brackets).*

We then calculated the wGRS of each MS and HC individual and compared the distribution of this variable between the two groups. In each model, MS patients had a higher wGRS than HC (figure 2 and table 2). The median wGRS of MS vs HC were 1.09 vs 0.00 (when considering only *HLA-DRB1*), 9.14 vs 8.15 (when considering *HLA-DRB1* + old SNPs) and 13.47 vs 12.46 (when considering *HLA-DRB1* + all known SNPs). The difference in wGRS between MS and HC was statistically significant in all considered models (table 2). We next assessed the predictive performance of each model using ROC curves and tested whether this has been considerably influenced by the discovery of additional MS genetic associations. The *HLA-DRB1* was on its own moderately able to discriminate between MS patients and HC (area under the curve (AUC) = 70.8% (95%CI = 63.4%–78.2%)). The AUC progressively increased to 76.6% (95%CI = 69.1%–84.2%) and to 79.7% (95%CI = 72.4%–87.0%) when including old SNPs and all currently known SNPs in the model respectively (figure 3). In the full model, the best wGRS threshold (i.e. the one providing the best sum of sensitivity + specificity) was 13.0 and this corresponded to a sensitivity of 71.4% and a specificity of 78.5% (figure 3). The AUC of models excluding *HLA-DRB1* and considering either old or all SNPs were 66.0% (95%CI = 57.2%–74.8%) and 69.3% (95%CI = 60.9%–77.8%) respectively.

The difference between predictive performances was significant between the model considering only *HLA-DRB1* vs the one considering *HLA-DRB1* + all SNPs (*p* = 0.016), while *HLA-DRB1* vs *HLA-DRB1* + old SNPs and *HLA-DRB1* + old SNPs vs *HLA-DRB1* + all SNPs trended towards significance (*p* = 0.054 and *p* = 0.13 respectively). However, when the prevalence of MS in the general population was considered (approximately 1/1,000), the positive predictive value of the full model was below 1% (i.e. the probability of having MS given a wGRS>13.0) [12].

## Discussion

Our knowledge of the genetics of complex diseases has greatly advanced in the last few years and the development of genome-wide analyses has enabled the discovery of more than 100 common variants influencing the risk of MS located outside the MHC. It seems plausible that even more variants could be discovered by further increasing the sample size and the statistical power of GWAS. MS researchers should therefore ponder upon what we have learnt from genetic studies in MS and what can potentially be derived from future ones. GWAS data have been extremely useful in helping us to improve our understanding of MS aetiology and its immunological nature. For example, gene-ontology analyses interrogating the genes located within MS-associated genomic regions have shown a substantial overrepresentation of Immune-related processes [4], [13]. Similarly, the integration of GWAS and open chromatin data has demonstrated that MS associated variants are particularly active in CD4+ T helper, CD8+ cytotoxic T and B cells [14]. What remains unclear today is whether genetic data can be similarly useful in disease prediction.

We used summary genetic association statistics to estimate what proportion of the general population can be predicted to be at a significantly different risk of MS as compared to an average baseline profile. We found that these estimates are clearly influenced by the strength and the number of genetic variants included in the model. As expected, considering one single variant of strong effect (such as *HLA-DRB1*) identified three significantly different risk categories in the population that correspond to the three possible genotypes at this locus. The distribution of risk became more continuous across the population when a larger number of variants were included such as non-MHC SNPs known in 2011 before the ImmunoChip study and novel ImmunoChip associations. Notably, the full model indicated that most individuals in the general population (about 90%) are either at average or reduced risk of MS, 10% have a risk higher than average and an extremely small fraction demonstrates a substantially increased risk (e.g. more than 10 times the average risk). This is comparable with previous estimates [15].

We then assessed to what extent two independent groups of MS patients and HC differed in terms of their position within this risk distribution. We found that in all considered genetic models, more MS patients than HC were identified at either elevated or high risk of disease. However, the inclusion of non-MHC SNPs in the models did not increase the number of MS patients considered at risk higher than average and, even when all variants were considered, half of MS patients appeared at either average or reduced risk of disease.

When the wGRS of MS was used as a predictor of disease status using ROC, the predictive performance improved with the increasing number of discovered associations and the best wGRS threshold in the full model was associated with 71.4% sensitivity and 78.5% specificity. This was significantly different from the AUC obtained considering only the *HLA-DRB1* association and higher than that based on the variants known in 2011. The lack of significance in the comparison between the models considering *HLA-DRB1* + all associations and *HLA-DRB1* + old associations should be interpreted with caution given the relatively small sample size of this study and the *p* value trending towards significance (*p* = 0.13). Nevertheless, when the low prevalence of MS was taken into account, the positive predictive value was very low.

The main limitation of this study is represented by the small sample size of MS patients and controls that were genotyped. Although predictive performance estimates may be influenced by increasing the sample size, our results are overall comparable with those of previous studies [7]–[9]. For example, Isobe et al found that MS patients from multi-case families have a greater genetic risk score than sporadic cases, but the predictive power of this estimate of genetic risk was still very limited [9]. Taken together, these findings indicate that the bias towards a greater genetic score found in MS patients is consistent across different sets of genetic associations and can be found in a relatively small sample size of patients and controls such as the one used in this study. Furthermore, the recent discovery of additional MS associated genetic variants has improved our ability to discriminate between MS patients and healthy individuals. Despite this, genetic data are still unlikely to be useful on their own and in this form for disease prediction in clinical settings.

A number of factors need to be considered for MS prediction. Several studies have implicated environmental agents in the aetiology of this disease and in particular a history of Epstein Barr virus infection, vitamin D deficiency and smoking [16]. Including these variables in the model may increase the predictive performance of the estimated risk. However, currently available data suggest that risk estimates are unlikely to be massively changed by considering the putative environmental agents in MS [15].

If neither genetics nor environment can fully predict disease development, then what determines MS onset? It is plausible that the effect of a risk factor on disease development is influenced by the presence or absence of additional agents. There is strong evidence for such mechanisms in classical monogenic conditions where the effect of mutations can be modified by epistatic interactions with other genetic variants and environmental factors [17], [18]. For example, the effect of phenylalanine hydroxylase mutations on the phenotype of phenylketonuria depends on dietary phenylalanine consumption [19]. Similarly, genetic variation on chromosome 21 has been reported to influence congenital heart defects in Down’s syndrome patients (all of whom have trisomy 21) [20]. It seems highly likely that similar mechanisms are at play in more complex diseases such as MS. Furthermore, environmental exposures both vary over time and are likely to act at specific time points; and it is often difficult (if not impossible) to take into account these factors in epidemiological studies [21], [22]. Environmental associations are therefore more difficult to measure with accuracy and this likely influences their potential role in disease prediction. A more full understanding of the complexity of MS, together with the interactions between risk factors and of the time specificity of environmental agents is needed to aid future attempts of disease prediction.

## Supporting Information

Table S1 **List of all currently known MS associated genetic variants.**
(XLSX)

Table S2 **Risk allele frequencies, Hardy-Weinberg equilibrium and proportion of missing genotypes** **for all MS associated genetic variants genotyped in MS patients and healthy controls.**
(XLSX)
